# Supplementary material for: Extensively Hydrolyzed Hypoallergenic Infant Formula with Retained T Cell Reactivity
Source: Nutrients. 2022 Dec 26;15(1):111. doi: 10.3390/nu15010111 (PMC9824366; doi:10.3390/nu15010111)
Supplement: Supplementary file 1 [file nutrients-15-00111-s001.zip › nutrients-1978614-supplementary.pdf]

Supplementary Materials:

Pilot experiment for determination of an allergen dose yielding basophil activation in the increasing part of the bell-shaped degranulation curve.

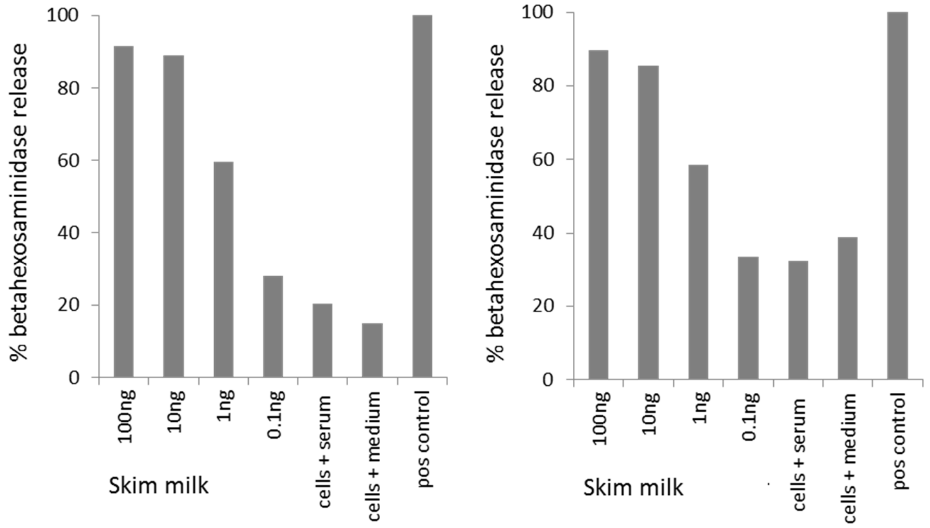

Figure S1. Pilot experiments determining the allergen dose inducing increasing basophil degranulation. Rat basophil leukemia (RBL) cells expressing the human FcεRI were loaded with serum IgE from a cow´s milk allergic patient (shown are two independent experiments (left, right) performed with serum IgE from patient #54. The percentages of released hexosaminidase as compared to total lysis (y-axes) by different concentrations of milk allergen extract (x-axes) are shown. Negative controls are cells+serum only, cells+medium only and a positive control was carried out with an unrelated control allergen (pos control).

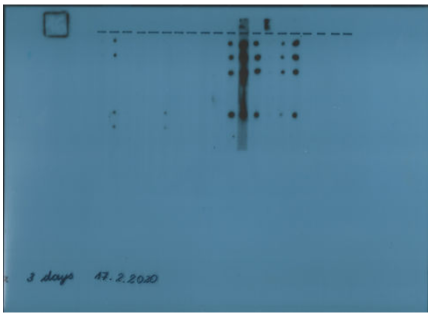

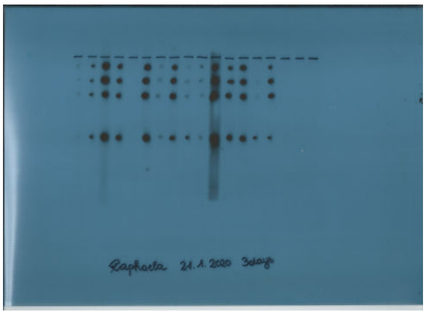

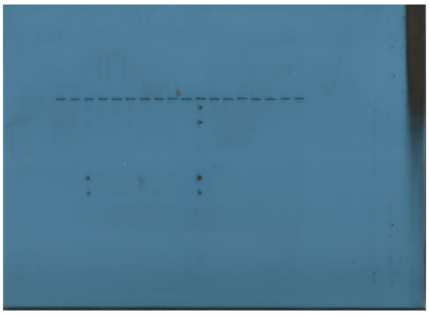

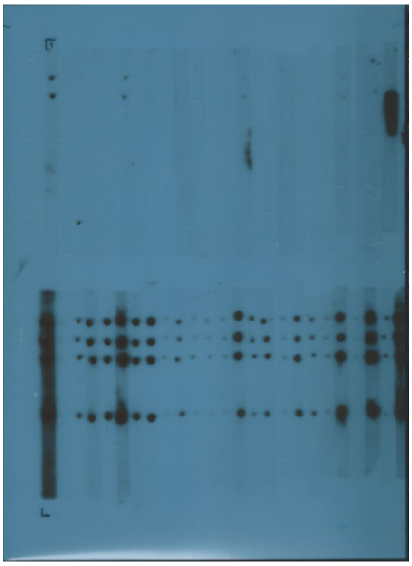

Figure S2. Original autoradiographs from Figure 2 without cropping.
